# Lymphoma in European hedgehogs (*Erinaceus europaeus*): A case series

**DOI:** 10.1177/03009858251367380

**Published:** 2025-09-04

**Authors:** Yannick Van de Weyer, Steve Bexton, Emanuele Ricci, Julian Chantrey, Valerie Tilston, Eva Dervas, Frauke Seehusen, Ana Gomez-Vitores, Liz Nabb, Hannah Kitchen, Hannah Tombs, Nick Woodger, Guido Rocchigiani

**Affiliations:** 1 University of Liverpool, Neston, UK; 2 RSPCA Stapeley Grange Wildlife Centre, Nantwich, UK; 3 RSPCA East Winch Wildlife Centre, King’s Lynn, UK; 4 University of Zurich, Zurich, Switzerland; 5 Animal Plant and Health Agency, Addlestone, UK; 6 Prickles and Paws, Newquay, UK; 7 Ashworth Veterinary Group, Farnborough, UK; 8 Finn Pathologists, Weybread, UK

**Keywords:** CD3, lymphoma, European hedgehog, hydrothorax, neoplasia, skin nodule, thymus

## Abstract

Neoplasia is rarely reported in European hedgehogs (*Erinaceus europaeus*). A retrospective search was conducted by contacting multiple veterinary diagnostic laboratories for cases of lymphoma in European hedgehogs. This resulted in 5 cases, from which clinical, gross, histologic, and immunophenotyping findings were recorded. Most animals (3/5) had skin masses involving the cervical region, 1 hedgehog had dyspnea and lethargy associated with hydrothorax, whereas another exhibited icterus and lethargy. The primary site of the lymphoma was the skin, particularly the neck or head (3/5), the thymus (1/5), and multicentric (1/5). Immunophenotyping confirmed B-cell lymphoma in 2 skin cases, a T-cell lineage for the thymic and multicentric cases, and undetermined for the remaining skin lymphoma. CD3, PAX5, and CD79a were reliable immunohistochemistry markers in formalin-fixed tissues in European hedgehogs. Although uncommon, lymphoma should be considered in the differential diagnosis for adult European hedgehogs with skin nodules, especially those seeming to originate from the neck.

Neoplasia is considered rare in European hedgehogs, accounting for 1.8% of all deaths according to one mortality study from Switzerland and Italy.^9^ This contrasts with pet African pygmy hedgehogs (*Atelerix albiventris*), which, likely due to widespread veterinary care and increased lifespan in captivity, more commonly present with neoplasias.^6^ This finding is likely multifactorial, with a comparatively lower average life expectancy in wild hedgehogs and a small percentage of carcasses presented for postmortem investigation, resulting in possible underestimation of neoplasia prevalence. Currently, mammary tumors represent the most described neoplasm in European hedgehogs,^4^ but this finding may be biased because mammary growths are often externally visible and, therefore, more easily detected. Various other neoplasms such as lymphoma, undifferentiated sarcoma, and carcinoma have been sporadically reported in European hedgehogs,^9,16^ but detailed descriptions are scarce.

Lymphomas are hematopoietic tumors arising from transformed lymphocytes and encompass a large group of different entities, with over 30 types described in dogs alone, all of which can have different biological behaviors and prognoses.^12,17^ Therefore, lymphomas in companion animals are generally classified according to the anatomical location of origin, size, and phenotype of the neoplastic lymphocytes, mitotic rate, and clinical behavior.^12^ Lymphoma is considered the second most common neoplasm in African pygmy hedgehogs, with gastrointestinal and multicentric forms predominating.^6^ One case of multicentric lymphoma has been reported in a greater hedgehog tenrec (*Setifer setosus*).^7^ Furthermore, sporadic cases of cutaneous T-cell lymphoma have also been reported in African hedgehogs^1,11^ but not in European ones. This case series describes lymphoma cases in European hedgehogs, providing clinical, macroscopic, histopathologic, and immunophenotypic information.

A retrospective search was conducted by consulting the University of Liverpool’s archives and by contacting colleagues active in the field of wildlife diagnostics for confirmed cases of lymphoma in European hedgehogs (University of Liverpool ethical approval reference VREC1550). This search yielded 5 cases (Table 1). Postmortem examinations were performed at the University of Liverpool (cases 1 and 3), East-Winch Wildlife Centre (case 2), the Animal and Plant Health Agency (case 4), and the University of Zurich (case 5). Histologic examination was performed on all major organs except for the central nervous system, which was unavailable for 2 animals (cases 2 and 4). For each external case, a set of unstained poly-lysine slides per representative paraffin-embedded tissue block was requested to perform in-house hematoxylin and eosin staining and immunohistochemistry (IHC). Aside from case 5, where initial CD3 IHC was performed by the submitting institution, a panel including CD3 (polyclonal rabbit, AO452, 1:500; Dako), CD79a (monoclonal mouse, MCA 2538H, 1:150; Bio-Rad), and PAX5 (monoclonal mouse, 610863, 1:100; BD Transduction Laboratories) markers was deployed for each case. An additional pancytokeratin IHC assay (monoclonal mouse, AE1/AE3, 1:200; Agilent) was used for case 1, to differentiate between thymic lymphoma and thymoma. IHC was performed using a Dako Autostainer Link 48. In brief, following heat-mediated antibody retrieval (high or low pH) and quenching of endogenous peroxidase, slides were incubated with primary antibody at room temperature before rinsing and adding labeled polymer Envision FLEX/HRP (dextran-coupled with peroxidase molecules and goat secondary antibody molecules against rabbit and mouse immunoglobulins; Agilent) for 20 minutes followed by visualization with diaminobenzidine chromogen with hematoxylin counterstain. Sections of normal lymphoid organs from European hedgehog’s showing evident lymphoid components (eg, lymph node) and canine lymph nodes (not shown) were used as positive controls, showing correct labeling patterns (Supplemental Fig. S1). As negative controls, we used the different tissues present in the same slides, which were unlabeled except for infiltrating T- and B-cells. CD20 and IBA1 IHC were also tested, providing no further aid in the lymphoma immunophenotyping (data not shown).

**Table 1.** Overview of lymphoma features in European hedgehogs (*Erinaceus europaeus*).

Case	Sex	Primary Site	IHC Phenotype	Lymphocytes Size	Mitotic Count
1	Male	Thymus	T-cell (CD3)	Small^a^ to intermediate^b^	1
2	Female	Skin (neck)	B-cell (CD79a)	Large^c^	11
3	Female	Skin (neck)	B-cell suspected (unusual PAX5)	Large^c^	9
4	Male	Skin (neck)	B-cell (PAX5)	Large^c^	1
5	Female	Spleen	T-cell (CD3)	Small^a^	1

Abbreviation: IHC, immunohistochemistry.

a Nucleus’ size of ~ 1 erythrocyte.

b Nucleus’ size of ~ 1.5 erythrocytes.

c Nucleus’ size of ≥ 2 erythrocytes.

European hedgehogs from this study were wild adult animals admitted to wildlife rehabilitation centers, 4 of which were in England and 1 (case 5) in Switzerland. Three were females and 2 males, ranging from 500 to 700 g in weight. Two animals were in poor body condition (cases 2 and 4), 1 (case 3) was in average body condition, and the remaining 2 (cases 1 and 5) were in good body condition. Case 1 presented for dyspnea and lethargy. Despite antibiotic therapy, the hedgehog developed anorexia, persistent dyspnea, and worsening lethargy, prompting veterinary attention whereby it died during transport to the nearest veterinary practice. Cases 2 to 4 presented with a single to multiple, firm, 1 to 3.5 cm diameter, soft tissue swellings affecting the cervical area and a history of being out during the daytime. In 2 animals (cases 2 and 4), nodules were ventrolateral, 1 of which (case 4) had concomitant purulent discharge, which led to the nodule being treated as an abscess with antibiotics and lancing. Both animals had progressive lethargy. Case 2 was euthanized for suspicions of neoplasia and clinical deterioration, whereas case 4 died overnight. Case 3 was initially bright and eating well, but it had a focal, ulcerated. 3.5 cm diameter mass dorsal to the cervical spine and extending to the cranium. Surgical excision was elected in this animal, and the resected tissue was submitted for histology, which suggested lymphoma with incomplete surgical margins. Within 3 weeks, pale tan to hemorrhagic, proliferative lesions were noted at the surgical incision site and oral cavity, after which the animal was euthanized. Case 5 presented with moderate icterus, hypothermia, lethargy, and a high ectoparasite burden. It died shortly after admission. Two hedgehogs (cases 2 and 3) were diagnosed antemortem with lungworm and treated accordingly.^8,13^

The postmortem examination of case 1 revealed clouded and moderately viscous, lymph-rich fluid within the thorax (chylothorax), which compressed the lungs (Fig. 1a). The cranial mediastinum was expanded by a 3 × 3 × 1.5 cm, multinodular, firm, trapezoidal mass. The pericardium was thickened with rare nodules measuring up to 0.4 cm in diameter and adhering to the mediastinal mass and parietal pleura. The lungs were diffusely, markedly atelectatic. Histologically, the cranial mediastinal mass was a moderately cellular, infiltrative, unencapsulated neoplasm composed of a biphasic cell population dominated by numerous small round cells, admixed with fewer cuboidal cells with large vesicular nucleus (Fig. 1b, c). The former cells were labeled for CD3, consistent with T lymphocytes (Fig. 1d), whereas the latter cell population was positive for pancytokeratin, consistent with thymic epithelial cells.^12^ Neoplastic lymphocytes were 1 to 1.5× the size of a red blood cell and characterized by a single round nucleus with heterochromatin, inconspicuous nucleoli, and scant to moderate amphophilic cytoplasm with distinct cell borders. Neoplastic T-cells were seen infiltrating the pericardium (Fig. 1b) and epicardium but not the lungs or abdominal organs.

**Figure 1.**
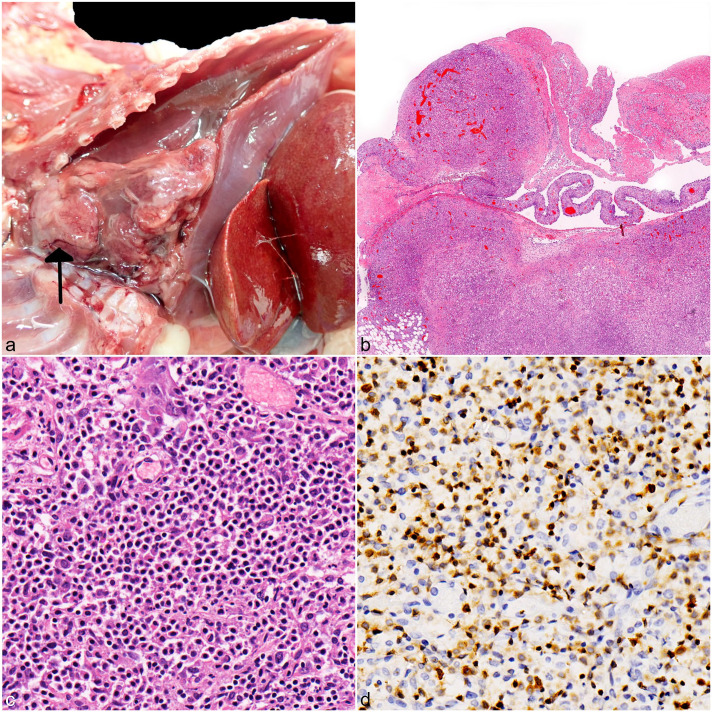
Gross, histologic, and immunohistochemical features of thymic lymphoma in European hedgehogs (*Erinaceus europaeus*). (a) Case 1, thoracic and abdominal cavities. A pale tan, multilobulated infiltrative mass is arising from the thymus (arrow) and occupies half of the thoracic cavity. The lungs are atelectatic due to chylothorax. The liver is congested with thin fibrin strands between adjacent lobes. (b) Case 1, thymus and pericardium. Multifocal, hypercellular nodular masses efface the thymus and pericardium, consistent with thymic lymphoma. Hematoxylin and eosin (HE). (c) Case 1, thymus. The mass is chiefly composed of small neoplastic lymphocytes together with fewer thymic epithelial cells. HE. (d) Case 1, thymus. Most neoplastic cells exhibit strong and membranous labeling for CD3. CD3 immunohistochemistry.

All hedgehogs with skin lymphoma (cases 2-4) had mild to moderate, diffuse hepatosplenomegaly on postmortem examination, whereas the hedgehog with multicentric lymphoma (case 5) had marked hepatosplenomegaly and lymphadenomegaly. Skin masses were variably pale tan (case 2), hemorrhagic (case 3), or cream (case 4) on cut-surface and had a rubbery texture. Metastases were present in all cutaneous lymphoma cases, and these were macroscopically characterized by relatively well-demarcated, variably sized, pale tan to hemorrhagic, rubbery masses in multiple visceral organs such as the liver, spleen, kidney, and adrenal gland (Fig. 2e, f). The skin or subcutaneous cervical lymph nodes were considered as the most likely primary lymphoma site for hedgehogs that presented with skin nodules, based on the size of the cervical mass (Fig. 2a). Histologically, the neoplasm in case 2 was located entirely in the subcutis, whereas both the subcutis and dermis were heavily effaced for cases 3 to 4. Neoplastic lymphocytes from 2 skin cases were characterized by intermediate to large cells with moderately vesicular chromatin, scant cytoplasm, marked cellular atypia, and a high mitotic count (~10 mitoses per 40× high-power field [0.152 mm^2^]) (Fig. 2b, c). Neoplastic cells from case 4 were also predominantly large but with dense heterochromatin, moderate amphophilic cytoplasm, and a low mitotic count (1 mitosis per high-power field). Lymph nodes of the skin cases were diffusely effaced by neoplastic lymphocytes with occasional breach beyond the capsule and complete loss of normal architecture. An immunohistochemical panel (CD3, PAX5, and CD79a) identified these skin neoplasms to be of B-cell lineage, but with inconsistent results for the B-cell markers. Neoplastic cells from case 2 exhibited moderate diffuse membranous immunoreactivity for CD79a (Fig. 2d), but they were negative for PAX5. Neoplastic cells from cases 3 and 4 were negative for CD79a, and case 4 exhibited mild diffuse nuclear labeling to PAX5. However, contrary to the positive control, PAX5 immunolabeling for case 3 appeared cytoplasmic rather than nuclear, which was unexpected. As such, artefactual PAX5 labeling cannot be excluded for this case, but histomorphology was nevertheless strongly suggestive of lymphoma. Histological examination of the multicentric case identified small neoplastic round cells with dense heterochromatin and scant to moderate cytoplasm within the liver and spleen, which was compatible with a T-cell lymphoma based on specific CD3 membranous immunoreactivity. In addition, case 5 had moderate chronic suppurative splenitis, mild lymphohistiocytic verminous pneumonia, mild chronic interstitial nephritis, and mild acute suppurative myocarditis. The icterus observed in this case was most likely secondary to the metastasis present in the liver.

**Figure 2.**
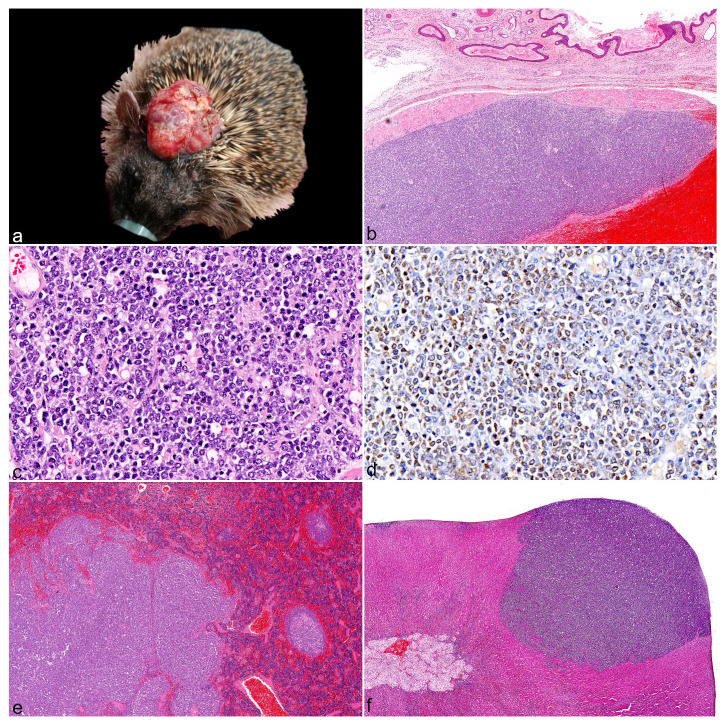
Gross, histologic, and immunohistochemical features of skin lymphomas in European hedgehogs (*Erinaceus europaeus*). (a) Case 3, skin lymphoma. A 3.5-cm diameter ulcerated mass is present in the dorsal cervical/head region. (b) Case 2, subcutaneous lymphoma, cervical skin. A focally extensive, well-demarcated neoplastic mass is present above a large area of hemorrhage within the subcutis. Hematoxylin and eosin (HE). (c) Case 2, subcutaneous lymphoma, cervical skin. Higher magnification of (b) showing neoplastic cells composed of monomorphic large lymphocytes. HE. (d) Case 2, subcutaneous lymphoma, cervical skin. Neoplastic cells have widespread membranous labeling for CD79a. CD79a immunohistochemistry. (e) Case 2, spleen. Markedly irregular, enlarged white pulp with loss of follicular architecture, compatible with lymphoma metastasis. HE. (f) Case 2, adrenal gland. Focal, densely cellular, well-demarcated round cell population effacing the cortex and compatible with lymphoma metastasis. HE.

This case series represents the first clinicopathological and immunohistochemical study investigating lymphoma in wild European hedgehogs. Most primary tumors were in the skin of the neck and/or head, which suggests that these anatomical regions are predilection sites for lymphoma in the species. Head and neck lymphomas in humans represent a heterogeneous group of entities with variable clinical presentations that can broadly be classified according to nodal versus extranodal origin.^14^ The latter are often diffuse large B-cell non-Hodgkin lymphomas that manifest as heterogeneous extranodal growths arising from the skin, salivary glands, paranasal sinuses, ocular adnexa, mandible, maxilla, and Waldeyer’s ring.^14^ Determining the exact origin within the cervical region proved difficult for these hedgehog cases, given the extensive infiltration of neoplastic cells into adjacent tissues at the time of diagnosis. Although primary cutaneous lymphoma remains a possibility, neoplastic cells appeared confined to the subcutis for case 2, epitheliotropism was not observed, and lymphomas with skin involvement in these European hedgehogs were of B-cell origin, which is relatively rare in other species. In humans, dogs, and cats, primary cutaneous lymphoma is most frequently of T-cell lineage,^2,3,5,17^ similar to reported cutaneous lymphomas in pet hedgehogs.^1,11^ Albeit speculative, 1 hypothesis for development of lymphoma in the cervical region of European hedgehogs could be chronic antigen stimulation of the cervical lymph nodes associated with verminous tracheitis, which is relatively common in this species.^8,9,16^ An alternative hypothesis could be subcutaneous injection in the cervical region promotes neoplastic transformation because this is a popular site for drug administration, but it was not possible to determine whether animals from this study had previously been under human care.

A cutaneous or inspiratory chemical exposure might have to be considered as well as the cause of the lymphomas. In rural areas, chemical compounds (eg, herbicides, pesticides) are commonly used, and considering the carcinogenicity of some of them (eg, glyphosate),^15^ exposure might constitute a risk.^10^ Retroviral infection can predispose to lymphoma in certain domestic animal species,^12^ but no such viruses have been described in European hedgehogs.

A limitation of this study is the relatively small number of cases described, which may not necessarily reflect the exact prevalence and prevalent phenotype of spontaneous primary lymphomas in European hedgehogs. Nevertheless, a diagnosis of skin lymphoma accounted for 2 out of a total of 9 hedgehog skin masses submitted by 2 wildlife rescue centers (RSPCA, UK). Differential diagnoses of cutaneous masses included granulomatous dermatitis, botryomycosis, soft tissue sarcoma, and mammary carcinoma (unpublished observation, YVdW). Therefore, additional cases should be reported and documented to improve our understanding and engage in improved detection and treatment protocols. Postmortem examinations were performed at different institutions, and bone marrow was often not available for histological interpretation, which hampered differentiation with leukemia and assessment of bone marrow invasion. Moreover, molecular markers and polymerase chain reaction (PCR) for antigen receptor rearrangement have not been established yet in this species, reducing diagnostic accuracy. Hedgehogs presented at an advanced state of disease with visceral metastases and poor prognosis. Theoretically, complete surgical excision may be curative when diagnosed at an early stage, especially for the cutaneous forms. However, European hedgehogs affected by lymphoma may not be presented for rehabilitation until after systemic spread has occurred, at which stage euthanasia is likely imminent because chemotherapy is currently not applied and is controversial in wild hedgehogs. Considering that all the skin lymphomas had metastasized to liver, spleen, and lymph nodes, a lympho-hematogenous spread seems the most plausible.

In conclusion, this case series describes spontaneous lymphoma in 5 European hedgehogs. Most cases had cervical skin involvement, which may be an anatomical region predisposed to developing lymphoma in this species. Hence, lymphoma should be considered part of the differential diagnosis in adult hedgehogs presenting with skin masses arising from local lymph nodes, the subcutis, or dermis of the neck and/or head. Animals from this study presented at an advanced stage, and the prognosis was poor. The T-cell marker CD3 seemed reliable for diagnostic purposes in these hedgehogs, but the B-cell marker PAX5 had variable results, probably due to the combined effect of incomplete antibody cross-reactivity and antigen-specific susceptibility to postmortem changes, which is why an IHC panel is recommended when B-cell lymphoma is suspected. Nonetheless, these IHC markers seem to perform well in control formalin-fixed tissues (Supplemental Fig. S1), dispelling doubts on the specificity of these markers in European hedgehogs.

## Supplemental Material

sj-pdf-1-vet-10.1177_03009858251367380 – Supplemental material for Lymphoma in European hedgehogs (Erinaceus europaeus): A case series Supplemental material, sj-pdf-1-vet-10.1177_03009858251367380 for Lymphoma in European hedgehogs (Erinaceus europaeus): A case series by Yannick Van de Weyer, Steve Bexton, Emanuele Ricci, Julian Chantrey, Valerie Tilston, Eva Dervas, Frauke Seehusen, Ana Gomez-Vitores, Liz Nabb, Hannah Kitchen, Hannah Tombs, Nick Woodger and Guido Rocchigiani in Veterinary Pathology
