# Supplementary material for: Lymphoma in European hedgehogs (Erinaceus europaeus): A case series
Source: Vet Pathol. 2025 Sep 4;63(2):212–7. doi: 10.1177/03009858251367380 (PMC12882974; doi:10.1177/03009858251367380)
Supplement: sj-pdf-1-vet-10.1177_03009858251367380 – Supplemental material for Lymphoma in European hedgehogs (Erinaceus europaeus): A case series [file sj-pdf-1-vet-10.1177_03009858251367380.pdf]

Supplemental Materials

Lymphoma in European hedgehogs (*Erinaceus Europaeus*): A case series

Yannick Van de Weyer, Steve Bexton, Emanuele Ricci, Julian Chantrey, Valerie Tilston, Eva Dervas, Frauke Seehusen, Ana Gomez-Vitores, Liz Nabb, Hannah Kitchen, Hannah Tombs, Nick Woodger, Guido Rocchigiani

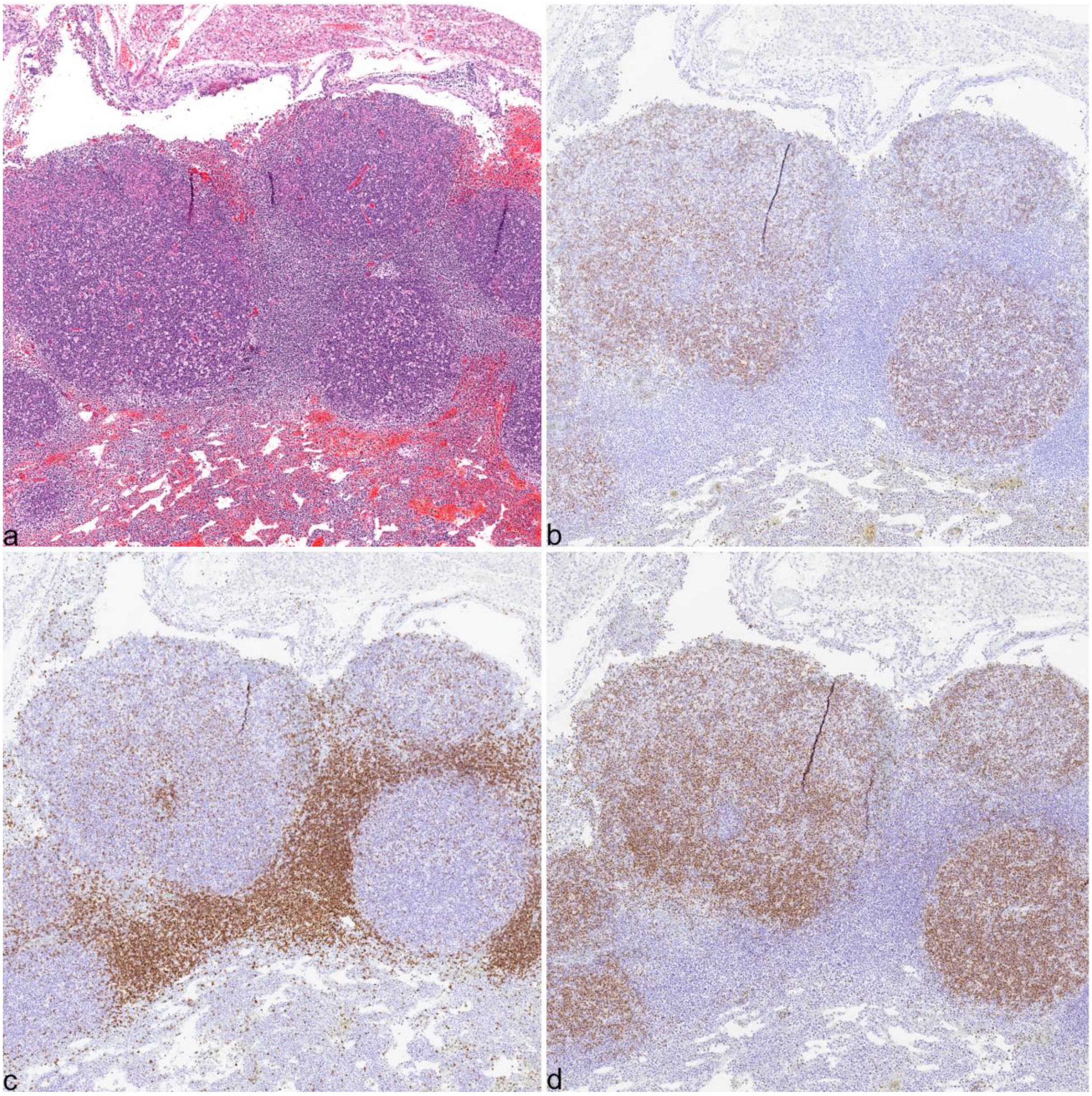

Supplemental Figure S1. Immunohistochemistry (IHC) of lymphoid markers on control lymph nodes of European hedgehogs (*Erinaceus europaeus*). a) Overview of a lymph node showing cortical lymphoid follicles. Hematoxylin and eosin. b) Lymph node. Most of the CD79a-labelled cells are localized in the lymphoid follicles and have membranous labelling. CD79a IHC. c) Lymph node. Most of the CD3-labelled cells are localized in the T zone, in between the lymphoid follicles, and have membranous labelling. CD3 IHC. d) Lymph node. Most of the PAX5-labelled cells are localized in the lymphoid follicles and have nuclear labelling. PAX5 IHC.
